# Development of repeatable arrays of proteins using immobilized DNA microplate (RAPID-M) technology

**DOI:** 10.1186/s13104-015-1637-3

**Published:** 2015-11-12

**Authors:** Nur Suhanawati Ashaari, Suganti Ramarad, Dzulaikha Khairuddin, Nor Azurah Mat Akhir, Yuka Hara, Nor Muhammad Mahadi, Rahmah Mohamed, Sheila Nathan

**Affiliations:** Malaysia Genome Institute, 43000 Bangi, Selangor DE Malaysia; School of Biosciences and Biotechnology, Faculty of Science and Technology, Universiti Kebangsaan Malaysia, 43600 Bangi, Selangor DE Malaysia; Xynergen Sdn. Bhd., UKM Technology Centre, 43600 Bangi, Selangor DE Malaysia; INTI International University, Bandar Baru Nilai, 71800 Nilai, Negeri Sembilan Malaysia

**Keywords:** Cell-free expression, Microarray fabrication, Protein array, RAPID-M

## Abstract

**Background:**

Protein microarrays have enormous potential as in vitro diagnostic tools stemming from the ability to miniaturize whilst generating maximum evaluation of diagnostically relevant information from minute amounts of sample. In this report, we present a method known as repeatable arrays of proteins using immobilized DNA microplates (RAPID-M) for high-throughput in situ protein microarray fabrication. The RAPID-M technology comprises of cell-free expression using immobilized DNA templates and in situ protein purification onto standard microarray slides.

**Results:**

To demonstrate proof-of-concept, the repeatable protein arrays developed using our RAPID-M technology utilized green fluorescent protein (GFP) and a bacterial outer membrane protein (OmpA) as the proteins of interest for microarray fabrication. Cell-free expression of OmpA and GFP proteins using beads-immobilized DNA yielded protein bands with the expected molecular sizes of 27 and 30 kDa, respectively. We demonstrate that the beads-immobilized DNA remained stable for at least four cycles of cell-free expression. The OmpA and GFP proteins were still functional after in situ purification on the Ni–NTA microarray slide.

**Conclusion:**

The RAPID-M platform for protein microarray fabrication of two different representative proteins was successfully developed.

## Background

Protein microarray technology has made enormous in-roads over the last decade and has become increasingly important as a research tool for proteomics studies and the development of new diagnostic platforms. Functional protein microarrays are being applied in several research areas including protein expression profiling, cellular signaling pathway mapping [1], interactome mapping [2], biomarker discovery [3] and systems biology [4]. Protein microarray technology allows parallel analysis of many proteins in a miniaturized format through a single experiment making the technology time and cost-effective [5].

Despite promising recent progress, protein microarray technology still has a number of technical challenges to overcome in order to achieve its maximum utility. Major challenges in protein arraying include the ability to express and purify a large number of proteins for array construction and to maintain protein folding and function in an immobilized state over long periods of storage [6]. Traditionally, the production of protein arrays has relied on cellular expression, purification and immobilization of individual proteins onto solid supports. This pipeline is a laborious and time-consuming process that inevitably presents a fresh set of technical challenges. To address these problems, several platforms have been developed that use cell-free systems and immobilized DNA to create protein arrays such as Nucleic Acid Programmable Protein Array (NAPPA) [7], Protein in situ Array (PISA) [8], DNA Array to Protein Array (DAPA) [9], *E. coli* Tus protein to Ter (TUS-TER) microarray [10] and microfluidic protein interaction network generator (PING-chip) [11]. These methods are also recognized as in situ cell-free expression protein microarrays where the protein arrays are generated from DNA fragments containing a C or N terminal fusion tag immobilized onto a solid support and coupled with a cell-free expression system [12]. Nevertheless, these described platforms have a number of limitations. For example, when using NAPPA and DAPA, the spot morphology of the arrays is often non-uniform due to protein diffusion during in situ protein synthesis [12] although this problem has been overcome with the development of the PING-chip [11]. The protein diffusion may often result in an overlap between neighbouring protein spots leading to difficulties in interpreting the array output data [9]. In addition, the NAPPA technology does not generate a pure protein microarray but instead is a mixed array in which proteins are co-localized together with the corresponding DNA template and capture antibody [13].

To address these limitations, we developed a new method known as repeatable arrays of proteins using immobilized DNA microplates (RAPID-M) based on immobilized DNA and cell-free expression technology. The RAPID-M technology was developed using 96-well PCR plates, which is useful for high throughput applications where 96 different proteins can be expressed simultaneously. The principle behind the RAPID-M technique is immobilization of the target gene onto beads to be used as template for cell-free expression to generate recombinant proteins that constitute the array. This approach provides numerous advantages over the previously described platforms as DNA immobilization and cell-free expression occurs in one single tube and the bead-immobilized DNA can be reused several times as template for cell-free expression. With other in situ cell-free expression protein microarrays, DNA is immobilized on the microarray slide or in the well. However the beads utilized in the RAPID-M system provide a larger surface area that permits relatively large amounts of DNA to be immobilized on the bead surface. RAPID-M generates multiple pure protein arrays that are separated from the DNA template making it an improvement over the NAPPA method that can only be used once as both protein and DNA are present together.

Here we provide proof-of-concept that the RAPID-M technology is suitable for protein microarray fabrication using Green Fluorescent Protein (GFP) and *Burkholderia pseudomallei* outer membrane protein A (OmpA) as our test proteins. GFP is widely used in biological and medical research as structure-wise, it is a stable molecule [14]. He and Taussig [8] and He et al. [9] also used GFP to demonstrate the principle of PISA and DAPA for protein microarray development. In addition, Angenendt et al. [15] developed a platform that combined nanowell chip technology and cell-free protein expression using GFP and β-galactosidase as their proteins of choice. The recombinant *B. pseudomallei* OmpA protein was successfully expressed in *E. coli* by Hara et al. [16] and shown to be highly suitable as a serodiagnostic antigen for the tropical disease melioidosis using different diagnostic formats [17]. *B. pseudomallei* is the causative agent of melioidosis and generally, diagnosis is fully dependent on bacterial culture. Many diagnostic tests have been developed to detect *B. pseudomallei* including real time polymerase chain reaction (PCR) detecting type III secretion systems [18], loop-mediated isothermal amplification (LAMP) method [19], multiple-antigen enzyme-linked immunosorbent assay (ELISA) [20] and a protein microarray [17]. However, none of these tests have been translated into a standard form of diagnosis. In this study, we chose *B. pseudomallei* OmpA protein as a representative bacterial protein along with GFP to demonstrate that the RAPID-M platform is suitable for protein microarray fabrication.

## Methods

### DNA templates

The pIVEX2.3d-GFP plasmid (5Prime, Germany) and linearized template of the outer membrane protein A (OmpA) (BPSL2522) encoding gene were used as DNA template. The pIVEX2.3d-GFP contains elements required for cell-free expression such as T7 promoter, ribosomal binding site, target gene encoding the 30 kDa wild-type Green Fluorescent Protein, C-terminal hexahistidine (6xHis) tag and T7 terminator.

The *ompA* gene encodes for *B. pseudomallei* OmpA outer membrane protein (27 kDa) [16]. The *ompA* linear template was generated using the RTS *E. coli* Linear Template Generation Set with a 6xHis-tag (5Prime, Germany). Linear templates of *ompA* for cell-free expression were generated through two PCR amplification reactions. The first PCR reaction amplified the *ompA* open reading frame. The *ompA* was amplified from *B. pseudomallei* genomic DNA using gene-specific sense (5′-CTTAAGAAGGAGATATACCATGAATAAACTTTCAAAG-3′) and antisense (5′-TGATGATGAG AACCCCCCCTTACTGCGCCGGAACGGTCG-3′) primers under the following reaction conditions: 500 ng *B. pseudomallei* genomic DNA, 1X Expand High Fidelity buffer without MgCl_2_, 3 mM MgCl_2_, 250 µM PCR Nucleotide Mix, 200 nM *ompA* sense and antisense primers, 3 U Expand High Fidelity Enzyme mix (5Prime, Germany) and H_2_O to a final volume of 50 µl. The PCR programme was set to an initial denaturation at 94 °C for 4 min, followed by 20 cycles of 94 °C for 1 min, 50 °C for 1 min, 72 °C for 1 min and final extension at 72 °C for 7 min. A second round of amplification was performed to add the T7 promoter and terminator regulatory elements and C-terminal 6xHis-tag to the *ompA* ORF by assembly PCR under the following conditions: 150–300 ng PCR product, 1X Expand High Fidelity buffer without MgCl_2_, 3 mM MgCl_2_, 250 µM PCR Nucleotide Mix, 480 nM T7 Promoter Primer (5Prime, Germany), 480 nM T7 Terminator Primer (5Prime, Germany), C-terminal 6xHis-tag DNA, 3 U Expand High Fidelity Enzyme mix and H_2_O to a final volume of 50 µl. The PCR was conducted using the following programme: initial denaturation at 94 °C for 4 min, followed by 20–25 cycles of 94 °C for 1 min, 60 °C for 1 min, 72 °C for 1 min and a final extension at 72 °C for 7 min.

### Oligonucleotide primers

The oligonucleotide primers were designed to anneal to the T7 promoter and terminator regions in order to amplify the target gene together with transcription and translation regulatory elements. The T7 promoter region primer (T7Prom, 5′-TAA TAC GAC TCA CTA TAG GGA GAC CAC-3′) and the T7 termination site primer (T7Term, 5′-TCC GGA TAT AGT TCC TCC-3′) were prepared and used as soluble primer and bead-immobilized PCR primer, respectively. The T7 terminator primer was modified with the addition of the amino (C12-NH_2_) or biotin (C12-Bt) group with a C12 carbon spacer at the 5′-end of the primer to enable primer immobilization onto the beads. The 12-carbon spacer was included to reduce steric hindrance between primer and beads.

### Immobilization of primers to beads

Dynabeads^®^ MyOne™ Carboxylic Acid (CA) (Invitrogen, NY, USA) and Dynabeads^®^ M-280 Streptavidin (SA) (Invitrogen, NY, USA) were used as the solid phases for primer immobilization. The CA and SA beads were used to immobilize the T7Term-NH_2_ primer and T7Term-Bt primer, respectively. The carboxylic group on the CA beads was activated with ethyl carbodiimide hydrochloride (EDC) for amide bonding with the primary amines (NH_2_) from the primer. A total of 5 nmol T7Term-NH_2_ modified primer is required per mg of CA beads. The mixture of CA beads, EDC and T7Term-NH_2_ primer were incubated at room temperature overnight. The T7Term-NH_2_ primer-immobilized beads (T7Term-NH_2_-CA) were then washed thoroughly with distilled water to remove any unbound primer. The SA beads were prepared by washing the beads with washing buffer [10 mM Tris HCl (pH 7.5), 1 mM EDTA, 2 M NaCl]. A total of 200 pmol T7Term-Bt primer was required to bind one mg of SA beads. The mixture of SA beads and T7Term-Bt primer was incubated at room temperature for 15 min with gentle rotation. The T7Term-Bt primer-immobilized beads (T7Term-Bt-SA) were then washed with washing buffer three times, followed by distilled water to remove any unbound primer.

### Solid phase PCR amplification

Solid phase PCR was initiated on both types of beads (T7Term-NH_2_-CA and T7Term-Bt-SA) following primer immobilization onto the beads. PCR reactions were conducted in 96-well PCR microplates and this format was adopted throughout the RAPID-M procedure. Standard PCR conditions were: 100–150 ng DNA template (pIVEX2.3d-GFP or *ompA* linearized template, 1 mM MgSO_4_, 1X PCR buffer, 0.3 mM dNTP mix, 0.8 pmol T7Prom soluble primer, 5 µg beads-immobilized primer and 1.25 U Platinum *pfx* DNA polymerase (Invitrogen) in a final reaction volume of 10 μl. A control reaction was run in parallel by replacing the beads-immobilized primer with 10 pmol of soluble T7Term primer.

The PCR programme involved an initial denaturation at 94 °C for 5 min, followed by 30 cycles of 94 °C for 1 min, 50 °C for 1 min and 72 °C for 1 min. The final cycle was followed by an additional 8 min at 72 °C to ensure complete extension. Subsequently, the supernatant was removed and the beads were washed thoroughly with distilled water to remove any unbound DNA. The beads-immobilized DNA templates were stored in 5 µl nuclease-free water until further use.

To confirm that the DNA template had been successfully immobilized onto the beads, a second amplification reaction was performed in a final volume of 50 μl reaction mixture containing 1 mM MgSO_4_, 1X PCR buffer, 0.3 mM dNTP mix, 0.8 pmol T7Prom and T7Term soluble primers, 1.25 U Platinum *pfx* DNA polymerase (Invitrogen, NY, USA), distilled deionized water and 5 μl beads-immobilized DNA. The PCR programme was carried out as described above.

### Cell-free expression

The beads-immobilized DNA was thoroughly washed and used as template for cell-free expression. For protein synthesis, the Rapid Translation System RTS 100 *E. coli* HY Kit (5 Primer, Germany) was used according to the manufacturer’s protocol. A total of 50 μl cell-free reagents were added to the same microwell plate containing the beads-immobilized DNA and incubated at 30 °C for 6 h with continuous agitation. At the end of the incubation, the plate was stored at 4 °C overnight to allow for protein maturation before the expressed proteins were spotted onto the microarray slide. A control reaction was done in parallel by replacing the beads-immobilized DNA with soluble DNA. The expressed His-tagged GFP and OmpA proteins were analyzed by Western blot using peroxidase-conjugated anti-polyHistidine monoclonal antibody (clone HIS-1) (Sigma Chemical Co., St Louis, USA).

### Fabrication of the protein microarray

The expressed recombinant GFP and OmpA proteins were printed onto nitrilotriacetic acid (Ni–NTA) slides (Xenopore, USA) using the Nano-Plotter™ NP 2.1 (GeSiM GmbH, Germany) equipped with piezoelectric Nano-Tips. Proteins were printed in nine separate spots with a centre-to-centre distance of 300 µm between spots and size of the individual spots was 200 µm. The arrays were incubated in a humidity chamber at room temperature overnight with 60–70 % humidity to provide efficient protein immobilization on the slide surface. Any unbound protein was removed with three washes with PBST buffer [0.01 M PBS buffer (pH 7.4), 0.05 % Tween-20 (Sigma)] of 10 min each. The nonspecific binding sites on the arrays were blocked with blocking buffer (5 % non-fat skim milk in PBST buffer) at 37 °C for 2 h with gentle rotation followed by washing with PBST buffer as above. The arrays were air-dried and stored dry at 4 °C until use.

### Probing

Anti-polyHistidine peroxidase-conjugated monoclonal antibody was diluted 1:2500 in blocking buffer and applied to the microarray fitted with a SecureSeal™ (Grace Bio-Labs, Bend, OR, USA). Incubation was performed in a humidity chamber at 37 °C for 2 h. The array was then washed three times with PBST buffer for 10 min each and the Tyramide Signal Amplification (TSA)-Cy5 system (Perkin Elmer, Boston, MA) was used to amplify the signal. The array was incubated with TSA-Cy5 at room temperature for 10 min. The slide was then washed three times with PBST buffer for 10 min each followed by distilled water and then air-dried. An InnoScan 700 scanner (Innopsys, France) was used for fluorescence detection of Cy5 and images were analyzed with the Mapix software package.

## Results and discussion

**Fig. 1:**
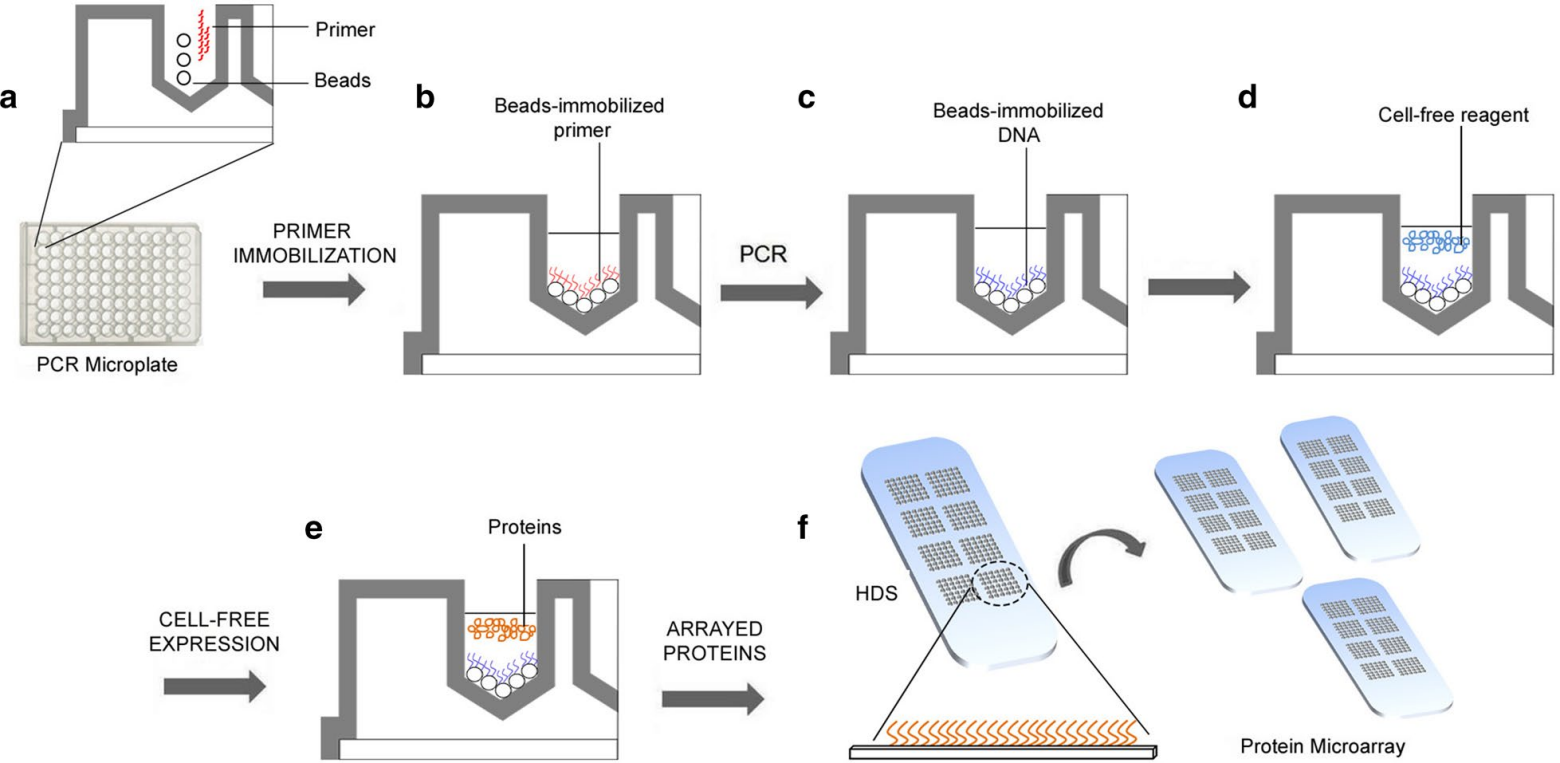
A schematic representation of the RAPID-M technology. The modified T7Term primer was mixed with the immobilization beads in a PCR microwell plate (**a**) to create beads-immobilized T7Term primer (**b**). The target DNA is directly immobilized to the beads by PCR amplification using beads-immobilized T7Term and soluble T7Prom to create high density immobilized DNA (HDID) in each well (**c**). The beads-immobilized DNA is translated to protein with the addition of cell-free reagents (**d**, **e**) and the protein is arrayed onto Ni–NTA slides using a microarray printer to produce high density protein microarray slides (HDS) (**f**)

During development of the RAPID-M platform, the modified T7Term primer was mixed with the immobilization beads (Fig. 1a) to create the beads-immobilized T7Term primer (Fig. 1b) in the PCR microwell plate. The target genes were immobilized directly onto the beads during PCR amplification using beads-immobilized T7Term primer and soluble T7Prom primer and this generated beads with a high density of immobilized DNA (HDID) within each well of the microplate (Fig. 1c). The beads-immobilized DNA was used as template for cell-free expression (Fig. 1d) to produce recombinant target proteins (Fig. 1e). These beads-immobilized DNA is reusable over many times without significant loss in efficiency. The expressed proteins are printed directly onto the glass slide to produce many identical protein arrays (Fig. 1f). Separate protein purification is not necessary in the RAPID-M approach as on-chip protein purification was incorporated onto the microarray slides. On-chip or in situ protein purification eliminates the need for resin-based purification and circumvents protein solubility and denaturation problems that occur during buffer exchange steps and freeze–thaw cycles [21].

### Solid phase PCR

Covalent linkage of DNA to the solid support provides increased stability of the attached DNA that is favourable for the subsequent hybridization steps. Similarly, the biotin-streptavidin system provides the strongest noncovalent interaction for DNA attachment [22]. We modified the 5′-end of the T7Term primer with either NH_2_ or the Biotin (Bt) group attached to CA and SA beads, respectively. The attachment of primer to beads should be at the 5-end of the primer to ensure that the 3′-OH end is available for DNA polymerase activity to initiate DNA amplification [23]. The beads-immobilized primer is elongated with the DNA polymerase to produce a copy of the immobilized DNA on the beads.

In designing the solid phase PCR experiments, two forms of DNA template were evaluated to demonstrate the versatility of the RAPID-M. The pIVEX2.3d plasmid containing the gene encoding wild type GFP and a linear template of the *B. pseudomallei ompA* represent the circular and linear forms of DNA, respectively. The DNA templates constructed for cell-free expression were composed of a T7 promoter, ribosomal binding site (RBS), C-terminal hexahistidine tag for protein immobilization and detection and the T7 terminator region. The incorporation of T7 promoter-driven expression in the RAPID-M approach would offer a universal platform for amplification and immobilization of target genes and protein expression. T7 promoter and T7 terminator have been widely used to express many recombinant proteins in cell-free expression systems and we envisaged that this method would work equally well for other bacterial genes.

We use the term solid phase PCR to describe PCR amplification with primers immobilized on the CA or SA beads. Solid phase PCR reactions were performed using CA beads and SA beads immobilized with 5′-T7Term-NH_2_ and 5′-T7Term-Bt primers, respectively. Once the primer immobilization step was achieved, both types of beads were subjected to PCR. The *gfp* and *ompA* genes were amplified from the pIVEX2.3d-GFP plasmid and linear template of *ompA*, respectively, using soluble T7Prom and beads-immobilized T7Term (5′-T7Term-NH_2_-CA beads or 5′-T7Term-Bt-SA beads). The reaction generated double stranded DNA on the beads by extension of the immobilized primers. Positive control reactions containing two soluble primers (T7Prom and 5′-T7Term-NH_2_ or 5′-T7Term-Bt) were set up in parallel.

**Fig. 2:**
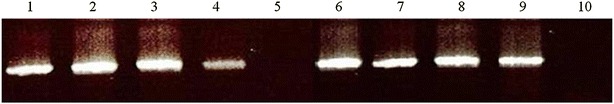
PCR amplification of *gfp* and *ompA* genes using T7Prom and T7Term primers. Primers for *gfp* amplification are as follows: *lane 1*, soluble T7Prom and 5′-T7Term-NH_2_ (as positive control); *lane 2*, soluble T7Prom and 5′-T7Term-Bt (as positive control); *lane 3*, soluble T7Prom and 5′-T7Term-NH_2_ with *gfp*-CA beads as template; *lane 4*, soluble T7Prom and 5′-T7Term-bt with *gfp*-SA beads as template; *lane 5*, non-template control (as negative control). Primers for *ompA* amplification as follows: *lane 6*, soluble T7Prom and 5′-T7Term-NH_2_ (as positive control); *lane 7*, soluble T7Prom and 5′-T7Term-Bt (as positive control); *lane 8*, soluble T7Prom and 5′-T7Term-NH_2_ with *ompA*-CA beads as template; *lane 9*, soluble T7Prom and 5′-T7Term-bt with *ompA*-SA beads as template; *lane 10*, non-template control (as negative control)

To verify the successful amplification and immobilization of double stranded DNA on the beads, a second PCR was carried out using beads-immobilized DNA from the first reaction, as the DNA template. The second amplifications for *gfp* and *ompA* were conducted using soluble T7Prom and T7Term primers. Figure 2 shows a representative amplification profile when *gfp* and *ompA* immobilized on CA and SA beads were used as the DNA template. Amplification of the *gfp* gene yielded a single band with the expected molecular size of 1121 bp for both the *gfp*-CA DNA template (Fig. 2, lane 3) and *gfp*-SA beads template (Fig. 2, lane 4). The *ompA* gene amplified from the *ompA*-CA beads template (Fig. 2, lane 8) and *ompA*-SA beads template (Fig. 2, lane 9) produced a single DNA band of 974 bp, as expected. The band intensity of the PCR products amplified from beads-immobilized DNA (Fig. 2, lanes 3, 4, 8, 9) is comparable to the amplified product from the soluble DNA template (Fig. 2, lanes 1, 2, 6, 7). This indicates that the beads-immobilized primer can be used to simultaneously amplify and immobilize the target gene from both a plasmid or PCR amplicon and concomitantly serve as DNA template for further amplification reactions. In contrast, Andreadis and Chrisey [22] reported that reactions containing soluble T7Prom and immobilized T7Term resulted in undetectable PCR product formation. The authors proposed that ‘spiking’ the reaction with soluble T7Term primer along with immobilized T7Term and soluble T7Prom increased the likelihood of interaction between DNA template and beads-immobilized T7Term primer. Nevertheless, in this study, we have successfully demonstrated that solid phase DNA amplification is possible using soluble T7Prom and immobilized T7Term primer without the need for additional soluble T7Term primer. The beads-immobilized DNA templates were subsequently utilised for cell-free expression to study the ability of the immobilized DNA to serve as template for cell-free protein production.

### Cell-free protein expression from beads-immobilized DNA template

**Fig. 3:**
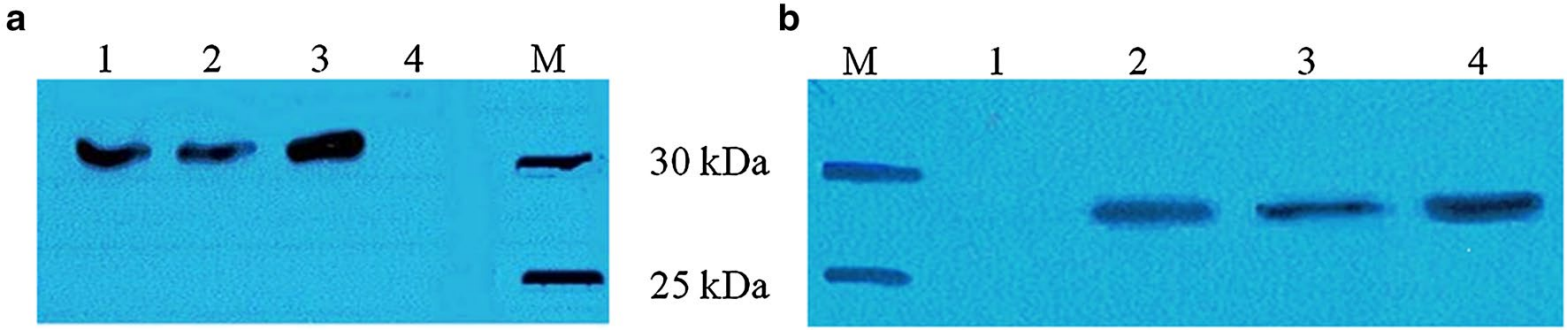
Western blot analysis of GFP (**a**) and OmpA (**b**) proteins using beads-immobilized DNA template. Proteins were detected using peroxidase-conjugated anti-polyHistidine monoclonal antibody. The calculated protein sizes for GFP and OmpA are 30 and 27 kDa, respectively. DNA templates used for cell-free expression were as follows: **a** *Lane 1*, *gfp*-SA beads template; *lane 2*, *gfp*-CA beads template; *lane 3*, soluble DNA coding for GFP (as positive control); *lane 4*, no DNA template (as negative control). **b** *Lane 1*, no DNA template (as negative control); *lane 2*, *ompA*-CA beads template; *lane 3*, *ompA*-SA beads template; *lane 4*, soluble ompA protein coding DNA (as positive control)

The advantage of a solid phase PCR amplification system using beads-immobilized DNA is the potential for repetitive use in cell-free expression reactions. The beads-immobilized DNA template provides a convenient means to express proteins by allowing the beads-immobilized DNA to be collected and recycled after each cell-free expression reaction. This approach eliminates the necessity for repeated DNA amplification and purification after every cell-free reaction, thus avoiding steps that are laborious and often expensive for high-throughput expression. In this cell-free expression experiment, the beads-immobilized DNA selected as templates for cell-free expression were *gfp*-CA beads, *gfp*-SA beads and *omp*A-CA beads. Western blot analysis showed that cell-free expression using beads-immobilized *gfp* and *ompA* genes produced protein bands with the expected molecular weight of 30 and 27 kDa, respectively (Fig. 3). This demonstrates that the beads-immobilized DNA was suitable as a template for cell-free expression.

**Fig. 4:**
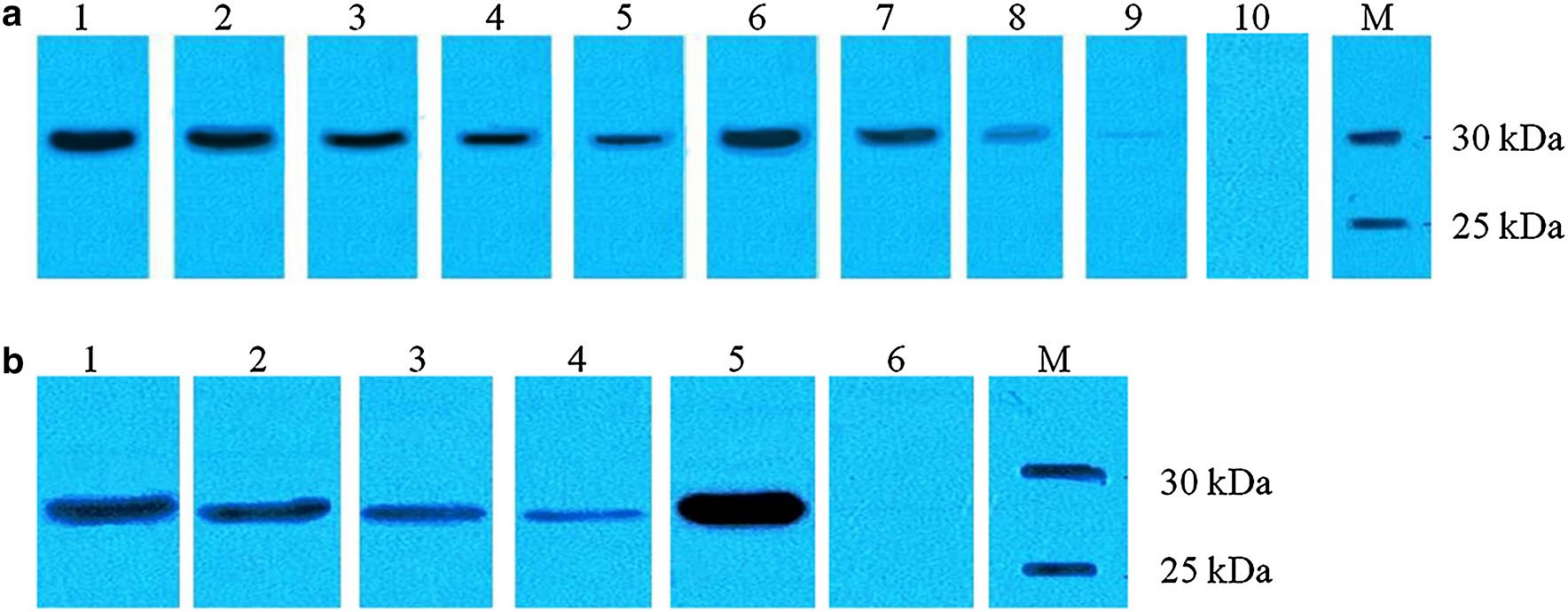
Repetitive cell-free expression of GFP and OmpA proteins using immobilized DNA templates. **a** *Lane 1*, GFP from soluble DNA (as positive control); *lanes 2–5*, GFP protein from the first, second, third and fourth sequential rounds of cell-free expression using *gfp*-CA beads template; *lanes 6-9*, GFP protein from the first, second, third and fourth sequential rounds of cell-free expression using *gfp*-SA beads template; *lane 10*, no DNA template (as negative control). **b** *Lanes 1–4*, OmpA protein from the first, second, third and fourth sequential rounds of cell-free expression using *ompA*-CA beads template; *lane 5*, OmpA protein from soluble DNA (as positive control); *lane 6*, no DNA template (as negative control)

The beads-immobilized *gfp* and *ompA* DNA were further analyzed to determine if they could serve as efficient templates for multiple sequential cell-free expression reactions. The beads-immobilized DNA were collected, washed and re-exposed to fresh cell-free reagent and this pipeline was repeated over four cycles. Our findings indicated that the beads-immobilized *gfp* and *ompA* templates were stable for at least four repetitive rounds of cell-free expression (Fig. 4). This demonstrates that the beads-immobilized DNA template can be used for repetitive cell-free expression without significant loss of expression efficiency. However, DNA immobilized to SA beads was not suitable for the repetitive cell-free expression where the expression of GFP molecules started to decrease in the third cycle of the repetitive cell-free expression (Fig. 4). On the other hand, we observed that the covalent immobilization between the NH_2_-terminal primer and CA beads was more suitable for repetitive cell-free expression. Our findings concur with that reported by Andreadis and Chrisey [24] who also noted that the biotin-avidin interaction is not suitable for repetitive cell-free expression. Hence, in this study, we have successfully expressed recombinant GFP and OmpA protein using immobilized DNA templates and these proteins were used for array fabrication in the next step of the RAPID-M method.

### Protein microarray fabrication

Development of the protein microarray platform based upon our RAPID-M approach incorporates in situ protein purification on metal chelate surface microarray slides as part of the process. The in situ protein purification on metal chelate chips synchronizes protein purification and immobilization, which significantly reduces, time, effort and cost for the post-protein expression steps [21]. In the RAPID-M approach, the expression system was designed to express recombinant proteins with a 6xHis-tag fused to the C-terminal end of the protein. The 6xHis-tagged fusion proteins were subjected to in situ purification and immobilization via specific interactions of the 6xHis-tagged proteins with a Ni^2+^ slide surface chelated by NTA. High affinity interaction of the 6xHis-tagged proteins with Ni^2+^ allows stringent washing conditions to remove the weakly bound protein impurities from the slide surface.

**Fig. 5:**
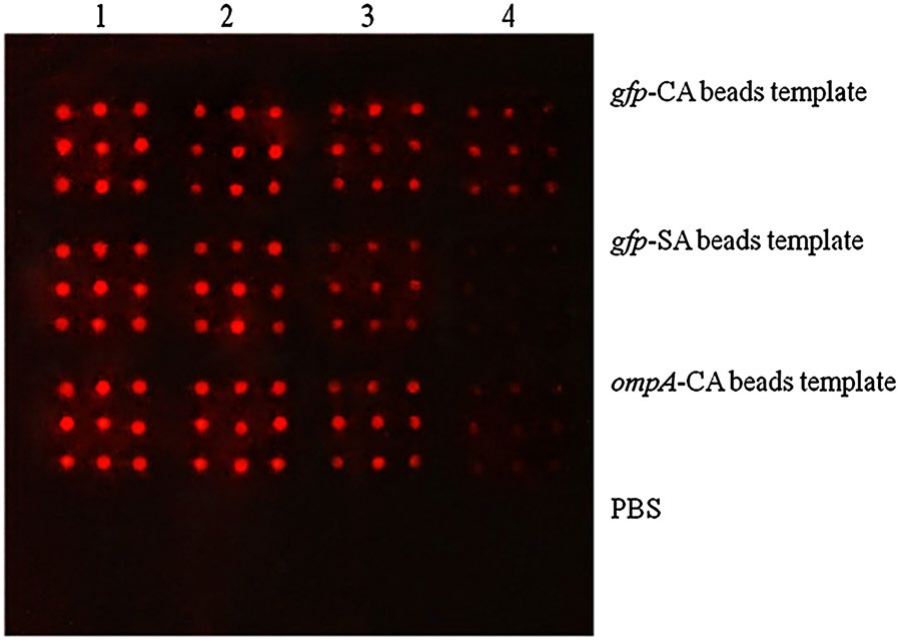
Application of the RAPID-M method for generation of protein arrays. The proteins were printed using the Nano-Plotter™ NP 2.1. Immobilized proteins were in situ purified on Ni–NTA chip and subjected to detection using peroxidase-conjugated anti-polyHistidine antibody followed by addition of TSA-Cy5. *Lane 1–4*, proteins from repetitive cycles of first, second, third and fourth rounds of cell-free expression using immobilized DNA template. Proteins are printed in 3 × 3 blocks and each block represents one cycle of cell-free expression using beads-immobilized DNA

Recombinant GFP and OmpA were still functional after in situ purification on the Ni–NTA surface as demonstrated in Fig. 5. The anti-polyHistidine peroxidase-conjugated antibody was able to recognize GFP expressed from the *gfp*-CA beads template up to the fourth round of cell-free expression. GFP expression from the *gfp*-SA beads template could only be detected until the third round of cell-free expression, which concurs with the earlier Western blot analysis (Fig. 4). The OmpA protein expressed from the CA beads template displayed a faint signal at the fourth round of cell-free expression. Thus, we conclude that we have successfully provided proof-of-concept of the utility of the RAPID-M platform for protein microarray fabrication.

Our purpose for developing the RAPID-M technology is to provide a versatile and flexible yet economical platform to fabricate protein microarrays. The RAPID-M approach makes high throughput protein production possible by combining cell-free expression technology with immobilized DNA templates and in situ purification. The use of immobilized DNA templates for cell-free expression without the need for DNA cloning provides a rapid means for translating genomic information into functional protein analysis. In contrast to conventional protein microarray fabrication where DNA templates are produced for each expression cycle, RAPID-M offers the ability to reuse the DNA template for cell-free expression. Hence, our proposed in situ protein purification and immobilization techniques pave the way for the generation of cost-effective protein microarrays without the need for cloning and post-expression purification. This platform also allows for the synthesized proteins to be directly spotted onto slides with uniform and consistent spot morphology. This is an advantage over the non-uniform spot morphology observed with NAPPA and DAPA [12] during in situ protein synthesis. Furthermore, the RAPID-M platform facilities the production of protein arrays on demand that would aid in overcoming issues pertaining to long-term storage of protein microarrays, particularly functional deterioration over time. Nevertheless, further optimization is necessary to improve the proposed model of the RAPID-M technology. This would include evaluating stability of the beads-immobilized template, analysis of target specificity and cross contamination issues. Assessing these parameters would be essential before the RAPID-M method can be utilized in the development of diagnostic protein arrays.

## Conclusions

Two DNA templates coding for the 30 kDa GFP and 27 kDa *B. pseudomallei* OmpA protein were immobilized to CA and SA beads. Both DNA templates immobilized to these beads were suitable to initiate cell-free expression. Nevertheless, we propose that the SA beads are not suitable for repetitive cell-free expression based on the significant decrease in protein expression after three cycles of expression on the reused template. In contrast, the covalent immobilization of DNA template onto CA beads holds promise for repetitive use of the immobilized DNA template for cell-free expression.

## Abbreviations

RAPID-M: repeatable arrays of proteins using immobilized DNA microplates
CA: carboxylic acid
SA: streptavidin
T7Prom: T7 promoter
T7Term: T7 terminator
bt: Biotin
NH2: Amino
NTA: nitriloacetic acid
HDID: high density immobilized DNA
HDS: high density protein microarray slides
